# P-1657. Antibiotics in hospice and the patients who receive them

**DOI:** 10.1093/ofid/ofae631.1823

**Published:** 2025-01-29

**Authors:** Patrick Crowley, Francis Whelan, Leslie Siegel, Douglas Challener

**Affiliations:** Mayo Clinic, Rochester, Minnesota; Mayo Clinic Rochester, Rochester, Minnesota; Mayo Clinic Rochester, Rochester, Minnesota; Mayo Clinic, Rochester, Minnesota

## Abstract

**Background:**

At the time of hospice enrollment, patients are asked whether they would potentially want antibiotics. This is little information regarding which patients are more likely to receive these antibiotics or the impact on comfort or longevity. To help guide discussions with patients and families and target future stewardship interventions, we sought to characterize the use of antibiotics in the outpatient hospice setting.

**Figure:**
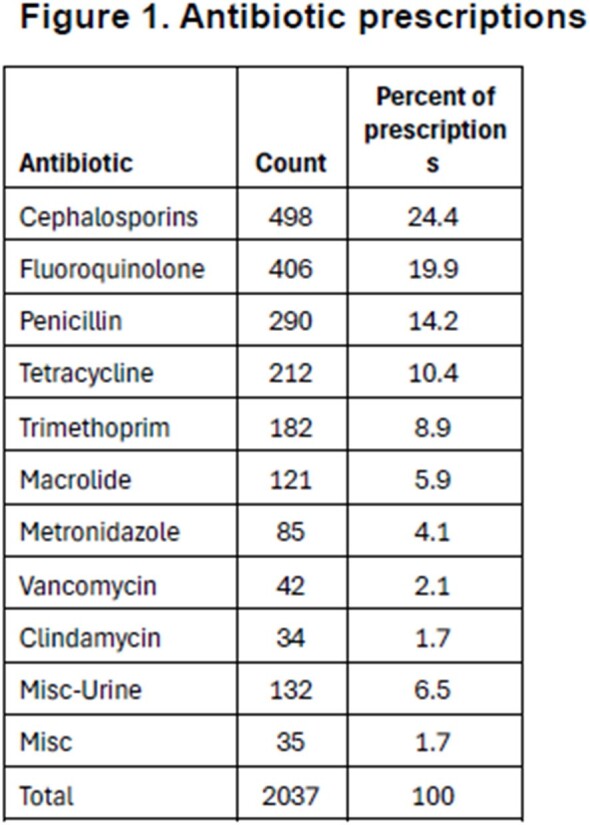

**Methods:**

We performed a retrospective review of patients enrolled in hospice with the Mayo Clinic Health System -Midwest from 1/1/2017 through 1/1/2023. For those with documented Hospice Qualifying Condition (HQC), we assessed age at enrollment, gender, survival time (ST), and Charleston Comorbidity Index (CCI). We documented which antibiotics were prescribed and compared the rate of prescription based on the above characteristics.

**Figure:**
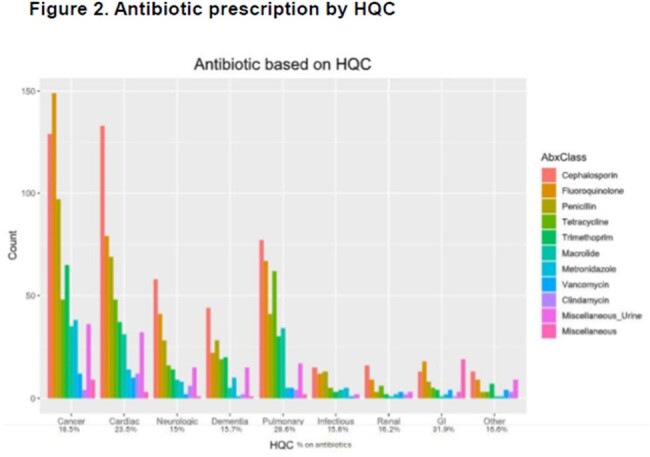

**Results:**

We identified 6452 patients with identifiable HQC. Of these, 1281 patients (19.8%) received 4173 antibiotic prescriptions. After removing refilled prescriptions, there were 2037 new prescriptions. Cephalosporins were the most common class of antibiotics prescribed (24.4% of antibiotics prescribed), followed by fluoroquinolones (19.9%), penicillin derivatives (14.4%), and trimethoprim (10.4%) (fig 1). Pulmonary (28.6%) and GI (31.9%) HQCs were most likely to receive antibiotics, neurologic was least likely (fig 2). There was no difference of age for those receiving antibiotics (80.6 yr) vs those not receiving (80.3 yr [p=0.53]), or for gender (male vs female OR 0.97 [0.86-1.10). CCI was higher in those patients who received antibiotics (4.3) than those not receiving (3.93, p< 0.001). Patients were more likely to receive antibiotics the longer they survived on hospice (4.7% surviving < 7d, 47.7% surviving >6mo – fig 3).

**Figure:**
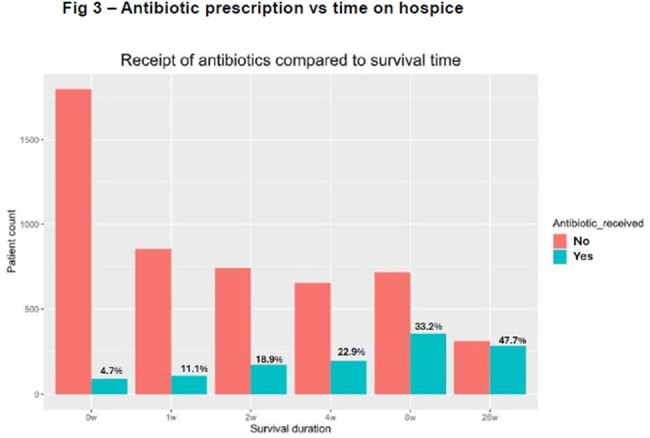

**Conclusion:**

Antibiotics are an important pharmaceutical tool in the hospice setting. Nearly 20% of patients in hospice will receive antibiotics during their hospice course, with higher chance in those surviving longer periods and those enrolled for GI or pulmonary reasons. Discussions of antibiotic are crucial for these groups, and antimicrobial steward efforts should consider focusing on these patients.

**Disclosures:**

**All Authors**: No reported disclosures

